# Different responses of soil bacterial community to plant–plant interactions under organic–inorganic fertilizers affect seedling establishment during subalpine forest succession

**DOI:** 10.3389/fmicb.2024.1466668

**Published:** 2024-10-01

**Authors:** Dandan Li, Yongping Kou, Jin Liang, Wenqiang Zhao, Dongdong Chen, Qing Liu

**Affiliations:** CAS Key Laboratory of Mountain Ecological Restoration and Bioresource Utilization & Ecological Restoration Biodiversity Conservation Key Laboratory of Sichuan Province, Chengdu Institute of Biology, Chinese Academy of Sciences, Chengdu, China

**Keywords:** bacterial community, plant–plant interactions, fertilization, forest succession, seedling establishment

## Abstract

**Introduction:**

Rhizosphere bacterial community as a valuable indicator of soil quality and function, has been widespread studied. However, little knowledge is about the response of bacterial communities to plant–plant interaction and different fertilizers during secondary forest succession.

**Methods:**

We conducted a field pot experiment applying organic and inorganic fertilizers to monocultures and mixed cultures of dominant plant species from mid- to late-successional stages (*Salix oritrepha*, *Betula albosinensis*, and *Picea asperata*), and investigated the responses of plant growth and rhizosphere bacterial communities.

**Results and discussion:**

Results indicated that growth rate of plant height varied among plant species, but no significant differences were observed in soil bacterial diversity and composition among plant species or inter-specific interactions under control. Compared to control, inorganic fertilizer resulted in increases in plant growth and the relative abundance of *Proteobacteria*, *Patescibacteria*, *Bacteroidetes* and *Gemmatimonadetes*, while simultaneously leading to decrease in the relative abundance of *Acidobacteria*, *Actinobacteria*, *Chloroflexi*, *Rokubacteria* and *Planctomycetes*. When grown with other species, the bacterial communities in the mixture resembled those of *S. oritrepha* in singular monoculture under inorganic fertilizer treatment, but plant growth was not affected by interspecific interaction. Unlike inorganic fertilizer, organic fertilizer significantly affected bacterial communities and increased bacterial diversity, but did not alter the effects of plant–plant interactions on bacterial communities. It was also observed that organic fertilizer facilitated later successional species’ growth (*P. asperata* and *B. albosinensis*) by the mid-successional species (*S. oritrepha*), ultimately facilitating secondary forest succession. In addition, plants at different successional stages harbor specific bacterial communities to affect their growth, and the bacterial communities contributed more than soil properties to the variations in the plant growth of *S. oritrepha* and *P. asperata* though the bacterial communities were regulated by soil factors. This finding highlights the significance of the rhizosphere bacteria on plant growth and plant community succession. It also emphasize the importance of considering both plant–plant interactions and diverse fertilizer types in forest restoration efforts and provide valuable insights into optimizing agronomic practices for secondary forest succession.

## 1 Introduction

The natural recovery of degraded forest in fact is a secondary forest succession process, where early-successional species are gradually replaced by late-successional species (Zhou et al., 2017; Liao et al., 2018). Secondary forest succession typically involves four successional stages: bare land, meadows, shrubs, and forest trees. The establishment of forest seedlings play a key role in determining whether or not degraded forest can successfully be restored into mature forests. Seedling establishment during secondary forest succession refers to coexistence of different dominant species across various successional stages. For example, during the secondary forest succession from shrub to forest, tree seedlings grow within shrub ecosystem and inevitably interact with shrubs. Therefore, the plant–plant interactions significantly influence the establishment of forest seedlings and play an important role in shaping plant communities and ecosystems (Guo et al., 2017; Urza et al., 2019).

Previous studies have indicated that the effect of plant–plant interaction can range from positive to negative or neutral as plants progress through different stages of their life-history (Baumeister and Callaway, 2006; Valiente-Banuet and Verdu, 2008; Paterno et al., 2016; Urza et al., 2019). The underlying mechanisms for these effects are diverse. Niinemets (2010) suggested that larger plants exhibit greater resource requirements and lower sensitivity to abiotic extremes. Maestre et al. (2005) made a meta-analysis and concluded that the net effects of plant–plant interactions did not increase with abiotic stress levels in arid and semiarid environments. More recently, there has been growing interest in studying the feedbacks between plants and soil microbes, emphasizing the great impact of interactions between plants and soil microbes on plant fitness, growth, and yield (Klironomos, 2002; Kardol et al., 2006; Teste et al., 2017). Mortimer et al. (2015) reported that a mixed tea species exhibited higher biomass of soil fungi and bacteria compared to a monoculture, resulting in increased productivity. And several studies have reported that soil microbes can influence plant–plant interactions by playing important roles in soil C/N/P cycles and nutrients availability to plants (Hendriks et al., 2015; Schimel, 2016; Liao et al., 2018). Given the great effects of soil microbes on plant–plant interaction and seedling establishment and a comprehensive understanding of the influence of plant interactions on soil microorganisms holding significant implications for forest restoration, it is necessary to investigate how soil microbes respond to plant–plant interaction during the process of forest seedling establishment in secondary forest succession.

Fertilization using organic and inorganic fertilizer is generally used to facilitate seedlings establishment. This practice plays an important role in nutrient cycling, plant health and soil properties. Previous studies have demonstrated that the application of inorganic N and P fertilizer enhances competitiveness among plants, with beneficial species exhibiting higher accumulations of C, N and P (Guo et al., 2017; Yu et al., 2019). Song et al. (2020) investigated two species during the early stage of primary succession under intra-and interspecific competition conditions with N fertilizer. They suggested that the plants with higher N uptake efficiency displayed greater competitiveness in intraspecific competition scenarios, and observed different composition of the soil bacterial communities in response to N fertilizer application and different competition patterns. And Pivato et al. (2017) found the effect of plants on bacterial community depended on N level. So far, these studies indicated that both plant–plant interaction and microbial communities are affected by inorganic fertilizer (Xia et al., 2016; Guo et al., 2019; Yu et al., 2019; Song et al., 2020). However, more and more research indicated that negative impact of inorganic fertilizer on environment and soil health (Zhang et al., 2015). Organic fertilizer has been proposed as an alternative approach for enhancing soil nutrition and reducing environmental impacts (Bebber and Richards, 2022). Although widely used in agriculture to improve crop yields, there is limited knowledge concerning their effects on forest recovery or soil bacterial communities within the forest ecosystem. Studying the impacts of different fertilizer types (inorganic and organic fertilizer) on the response of soil bacterial community to plant–plant interactions during forest seedlings establishment will enhance our understanding of underlying mechanisms governing the effects of plant–plant interactions and fertilizer management practices on soil bacterial diversity and composition, and thereby providing scientific and technological support for forest restoration.

The subalpine forest in the southwest China encountered extensive deforestation and grazing in the mid-20th century, as well as the combined effects of climate change, natural disasters and major anthropogenic activities such as the construction of roads, railways, and hydropower stations in recent years, resulting in the existence of large areas of secondary forest in this region. However, the process of natural restoration from secondary forest to zonal top vegetation forest is relative slow, usually takes several decades, so it is urgent to take artificial measures to promote vegetation restoration and ensure the ecological security of the region. The restoration of secondary forests in this region encompasses the middle and late successional stage of forests typically dominated by shrubs, broadleaf trees and conifers, in their respective ecological communities. Thus, we conducted a field pot experiment using organic and inorganic fertilizers and planted *Salix oritrepha* (shrub species), *Betula albosinensis* (broadleaf species), and *Picea asperata* (conifer species) seedlings in monocultures and mixed cultures to investigate changes of the rhizosphere bacterial communities and plant growth. We aimed to elucidate that (1) the impacts of different dominant plant species at various successional stages on rhizosphere bacterial communities; (2) whether interspecific interactions lead to rhizoshpere bacterial communities resembling those of the superior competitor conifer species in singular monoculture when grown with other species in mixture; and (3) the influence of inorganic and organic fertilizer on soil bacterial communities, as well as the responses of bacterial communities to plant–plant interactions. This study aims to reveal the underlying mechanisms affecting seedling establishment and plant growth strategies, ultimately promoting forest seedling establishment and facilitating secondary forest succession in this region.

## 2 Materials and methods

### 2.1 Plant species

To investigate differential effects of plant species and inter-specific plant–plant interactions among different secondary successional stage species, we selected *Salix oritrepha* as a representative middle-successional shrub specie, and late-successional species including *Betula albosinensis*, a broadleaf specie and *Picea asperata*, a conifer specie. The *Salix oritrepha* seedlings with a height of 40–45 cm were obtained from a local field shrub ecosystem while *Betula albosinensis* seedlings with a height of 45–50 cm were sourced from a nearby forest ecosystem. The *Picea asperata* seedlings with a height of 25–30 cm were acquired from a local nursery garden. In order to ensure uniformity in the rhizosphere soil of the all three species before the pot experiment and improve their survival rate, we first transplanted these seedlings to an adjacent *Picea asperata* plantation for acclimation over a period of 3 months before conducting the pot experiment.

### 2.2 Experimental design

The field pot experiment was conducted in an open field area near the Miyaoluo Natural Reserve of Lixian County, located on the eastern Tibetan Plateau, Sichuan Province, China (31°47′N, 102°42′E, 3210 m, a.s.l) with mean annual temperature 8.7°C, annual precipitation ranging from 600 to 1,100 mm, and annual evaporation ranging from 1,000 to1900mm. The soil is classified as mountain brown soil based on Chinese soil taxonomy. Soils were collected from nearby *Picea asperata* plantation and sieved with 4 mm mesh to eliminate roots and stones, and then were homogenized (pH 6.53, TOC 52.62 g/kg, TN 4.10 g/kg, available P 12.59 mg/kg, and available K 128.33 mg/kg). To investigate the differential effects of plant species and inter-specific plant interactions on soil bacterial communities, each two *Salix oritrepha* seedlings (SS), two *Betula albosinensis* seedlings (BB), and two *Picea asperata* seedlings (PP) as monocultures, and two combination of SB, SP, and BP seedlings as mixed cultures were planted 10 cm apart from each other into each plastic cylindrical pot (inner diameter 38 cm and depth 34 cm) in April 2018. Each combination of seedlings included 30 pots for applying inorganic and organic fertilizers, and control without fertilizer.

To examine the impact of fertilization on soil bacterial communities, we applied two types of fertilizer: organic and inorganic fertilizer. Each 10 randomly selected pots were used for each plant–plant interaction treatment to apply either organic fertilizer or inorganic fertilization, while 10 randomly selected pots were set up for controls without any fertilizer. Each treatment had 10 replicates. The organic fertilizer was derived from the local decomposed edible fungi residue, which was mixed with soil as a base fertilizer at the beginning of the experiment. A total of 60 pots with organic fertilizer were applied for 100 kg of organic fertilizer throughout the study period. This organic fertilizer contained water content 70%, N 3.78%, available K 3.91% and available P 0.71% of the dry mass. The inorganic fertilizer, consisting of KNO_3_, (NH_4_)_3_PO_4_, (NH_4_)_2_SO_4_, Ca(NO_3_)_2_, was dissolved in water and applied to the plant roots through watering once a month from May 2018 to September 2019 with equivalent amounts provided in each pot throughout the experiment to match the N, P, K content of the organic fertilizer. We regularly measured the plant height from 2 months after the start of experiment until its end.

### 2.3 Soil samples

We sampled rhizosphere soil twice with a bore (8 cm diameter), one in August 2018 at the beginning of the experiment and the other in October 2019 at the end of the experiment, respectively. These samples were subsequently sieved through a 2 mm mesh, stored in an ice box at 4°C, and transported to laboratory for analysis. One subsample was stored in −80°C for soil bacterial communities analysis while another subsample was air dried for the soil properties analysis.

### 2.4 Soil properties analysis

Soil pH was determined in suspending soil in water at a ratio of 1:2.5 v/v. The extraction method used for available phosphorus (AP) involved NaHCO_3_ extracts, following the procedure developed by Olsen et al. (1954). For available potassium (AK), ammonium acetate was used as an extracting agent, and the measurement was conducted using a flame spectrophotometer. Detailed methodologies to determine concentrations of soil organic C (SOC), dissolve organic C (DOC), total nitrogen (TN), ammonium (NH_4_^+^), nitrate (NO_3_^−^), and nitrite (NO_2_^−^)-N concentrations, as well as microbial biomass C (MBC) and N (MBN), were previously described in our study (Li et al., 2019).

### 2.5 DNA extraction and PCR amplification

Soil DNA was extracted from approximately 0.5 g of fresh soil sample using the OMEGA Soil DNA Kit (M5635-02) (Omega Bio-Tek, Norcross, GA, United States) following the manufacturer’s instructions. Subsequently, it was stored at −20°C until further analysis. The quantity and quality of the extracted DNAs were assessed using a NanoDrop NC2000 spectrophotometer (Thermo Fisher Scientific, Waltham, MA, United States) and agarose gel electrophoresis, respectively.

PCR amplification was conducted to amplify the bacterial 16S rRNA gene V3–V4 region using forward primer 338F (5ˊ-ACTCCTACGGGAGGCAGCA-3ˊ) and reverse primer 806R (5ˊ-GGACTACHVGGGTWTCTAAT-3ˊ). The PCR reaction mixture included 5 μL of buffer (5×), 0.25 μL of Fast pfu DNA Polymerase (5 U/μl), 2 μL (2.5 mM) of dNTPs, 1 μL (10 uM) of each Forward and Reverse primer, 1 μL of DNA Template, and 14.75 μL of ddH2O. The PCR process began with an initial denaturation step at 98°C for 5 min, followed by 25 cycles consisting of denaturation for 30 s at 98°C, annealing for 30 s at 55°C, and extension for 45 s at 72°C. Finally, a 5 min extension step was performed at 72°C. The resulting PCR amplicons were purified with Vazyme VAHTSTM DNA Clean Beads (Vazyme, Nanjing, China) and quantified utilizing the Quant-iT PicoGreen dsDNA Assay Kit (Invitrogen, Carlsbad, CA, United States). After the individual quantification step, amplicons were pooled in equal amounts, and paired-end 2 × 250 bp sequencing was performed using the Illlumina NovaSeq platform with NovaSeq 6,000 SP Regent Kit (500 cycles) at Shanghai Personal Biotechnology Co., Ltd. (Shanghai, China).

The sequencing data analyses were performed with QIIME2 (Bolyen et al., 2018) with slight modification according to the official tutorials.^1^ In order to minimize variations in sequencing depth across samples, an averaged and rounded rarefied ASV table was generated at an equal depth of 55,119 sequences per sample. Taxonomic classification of the ASVs was performed based on the Silva Release 132 Database.

### 2.6 Data analysis

Bioinformatics analyses were mainly performed using QIIME2 and R packages (v.4.4.0). The alpha diversity indices at ASV-level, such as Chao1 richness estimator, Observed species (Observed OTU) and Shannon diversity index, were calculated using the ASV table in QIIME2 and visualized as box plots. Principal Coordinate Analyses (PCoA) was performed visual the differences of bacterial community across samples based on the Weighted UniFrac distance metric. Permutational multivariate analysis of variance (PERMANOVA) method with 999 permutations was used to determine the effects of fertilizer and plant–plant interactions on the bacterial communities. Linear discriminant analysis effect size (LEfSe) was performed to detect differently abundant taxa across groups. Above microbial analyses were conducted by the genescloud tools, a free online platform for data analysis.^2^

The growth rate of plant height, representing the annual increase rate in plant height, was calculated as the ratio of the change in plant height from the beginning to the end of the experiment relative to the initial plant height at the start of the experiment, and is expressed as a percentage annual growth rate. The SPSS software was utilized to conduct an analysis of variance (ANOVA) in order to assess the significant differences among the factors such as fertilization, plant–plant interaction, and sample time. These factors were examined for their impacts on soil properties, growth rate of plant height, bacterial taxa abundance (specifically the top 10 of phylum with highest average abundance), as well as alpha diversity indices of bacterial communities. The redundancy analysis (RDA) was used to investigate the effects of environmental factors on the bacterial communities and alpha diversity indices. Pearson’s correlation in SPSS and linear regression in Origin 21 also were used to determine the relationship between relative plant growth and the relative abundances of top 20 bacteria taxa from phylum to genus. Variation partitioning analysis (VPA) with the “varpart” function in vegan package were performed in R (v.4.4.0) to depict the shared and independent effects of soil properties and bacterial community on the growth rate of plant height.

## 3 Results

### 3.1 Plant height growth rate

**Figure 1:**
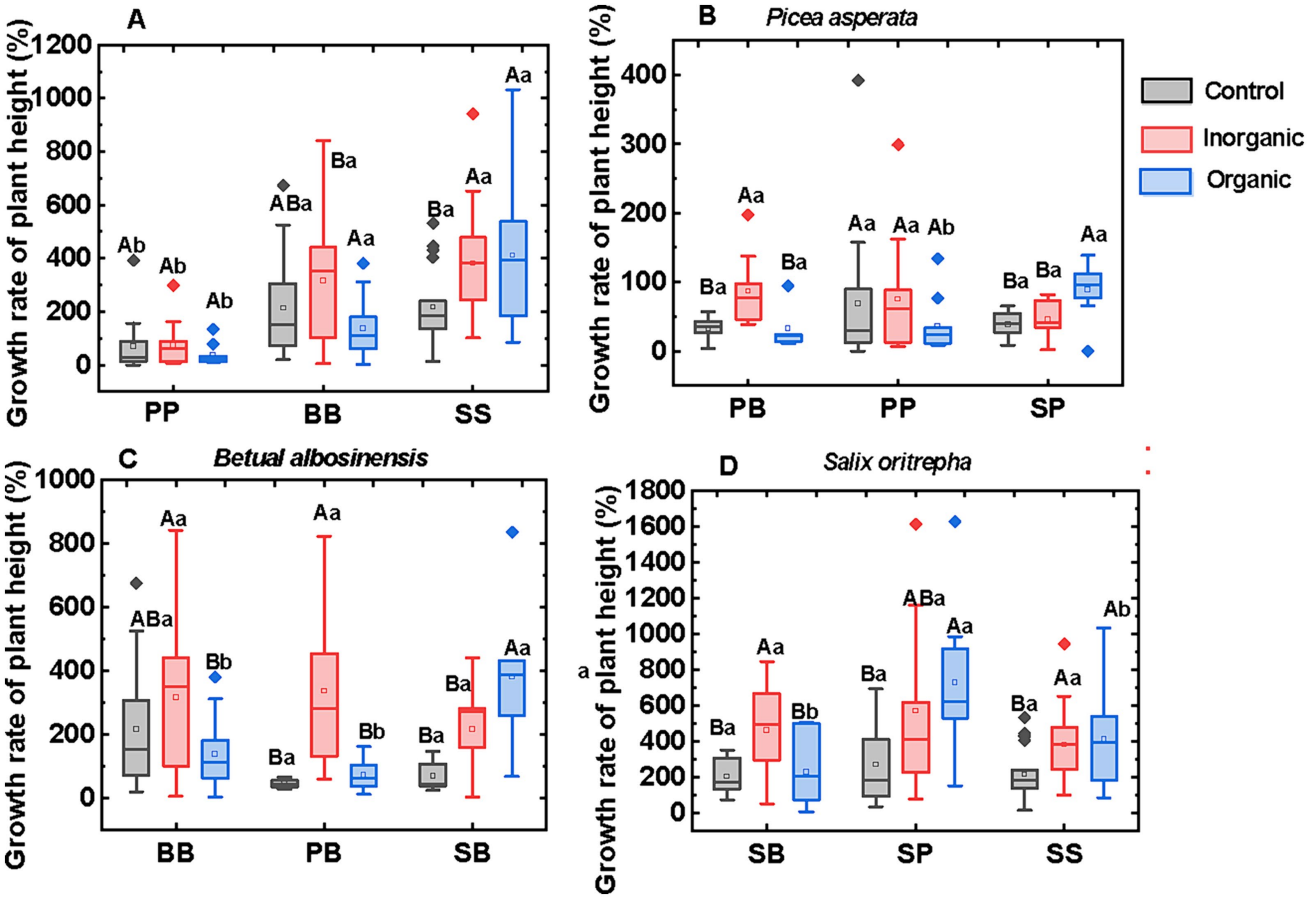
Annual growth rate of plant height across species **(A)** and interspecific interactions **(B–D)**. The capital letters above the column indicate significant differences across different fertilization treatments, while the lowercase letters above the column indicate significant differences across different plant–plant interactions. The capital letter B, S, and P represent plant broadleaf species *Betula albosinensis*, shrub species *Salix oritrepha*, and conifer species *Picea asperata*, respectively; BB, SS, and PP refer to intraspecific plant–plant interactions, while SB, SP, and BP refer to interspecific plant–plant interactions.

The growth rate of plant height varied with plant species across various fertilization treatments (Figure 1A). Growth rate of shrub seedlings (*S.oritrepha*) exhibited the highest among the three species, while conifer seedlings (*P.asperata*) displayed the slowest growth rate in all the fertilization treatments. The interspecific interactions only affected the growth rates of plant height in the organic fertilizer treatment, but not in the control and inorganic fertilizer treatment (Figures 1B–D). When *B.albosinensis* (B) and *P.asperata* (P) grew with *S.oritrepha* (S) in mixed culture, their growth rates were higher than in singular monoculture under organic fertilizer treatment. Furthermore, in the singular monoculture SS and mixed culture (SP and SB), organic fertilizer promoted their growths of all these plants compared to control. In addition, irrespective plant species or interspecific interactions, plants in the inorganic fertilizer treatment grew faster than in the organic fertilizer treatment and control.

### 3.2 Soil properties

The concentrations of soil DOC, NH_4_^+^, NO_2_^−^, available K, MBC, MBN and MBC/MBN were significantly affected by plant–plant interactions, particularly under fertilization treatment (Table 1, Supplementary Table S1). The significant differences of these soil properties resulting from plant–plant interactions were affected by the fertilization. The impact of fertilization on soil properties was more than that of plant–plant interactions. Both inorganic fertilizer and organic fertilizer increased the concentrations of soil NH_4_^+^, DOC, available P and available K, MBC and MBC/MBN but decreased the concentration of soil NO_2_^−^. Moreover, organic fertilizer had a greater effect on those soil properties than inorganic fertilizer. Additionally, while inorganic fertilizer decreased the soil pH, and MBN, the organic fertilizer increased them (Supplementary Table S1).

**Table 1:** Effects of fertilization and plant–plant interactions on soil properties (two-way ANOVA).

		pH	DOC	TOC	TN	TC/TN	NO_3_^−^	NH_4_^+^	NO_2_^−^	AP	AK	MBC	MBN	MBC/MBN
Fertilization	F	703.1	21.26	91.50	257.7	29.83	0.12	22.84	704.0	59.6	198.9	282.4	149.9	71.46
	P	**0.000**	**0.000**	**0.000**	**0.000**	**0.000**	0.891	**0.000**	**0.000**	**0.000**	**0.000**	**0.000**	**0.000**	**0.000**
Plant–plant	F	1.583	8.305	1.541	2.403	0.865	1.082	2.645	12.391	1.944	2.782	3.835	9.594	6.496
	P	0.190	**0.000**	0.202	0.056	0.514	0.387	**0.039**	**0.000**	0.111	**0.032**	**0.007**	**0.000**	**0.000**
Fertilizer*Plant	F	1.887	2.189	1.542	1.866	1.234	1.172	4.526	10.951	3.835	0.647	3.241	1.108	2.017
	P	0.080	**0.042**	0.165	0.084	0.304	0.341	**0.000**	**0.000**	**0.001**	0.764	**0.005**	0.383	0.061

DOC, dissolve organic carbon; TOC, total soil organic carbon; TN, total soil nitrogen; AP, available phosphorus; AK, available potassium; MBC, microbial biomass carbon; MBN, microbial biomass nitrogen. The bold values in table represent the significant difference between the factors analyzed by ANOVA test.

### 3.3 Soil bacterial community diversity

**Figure 2:**
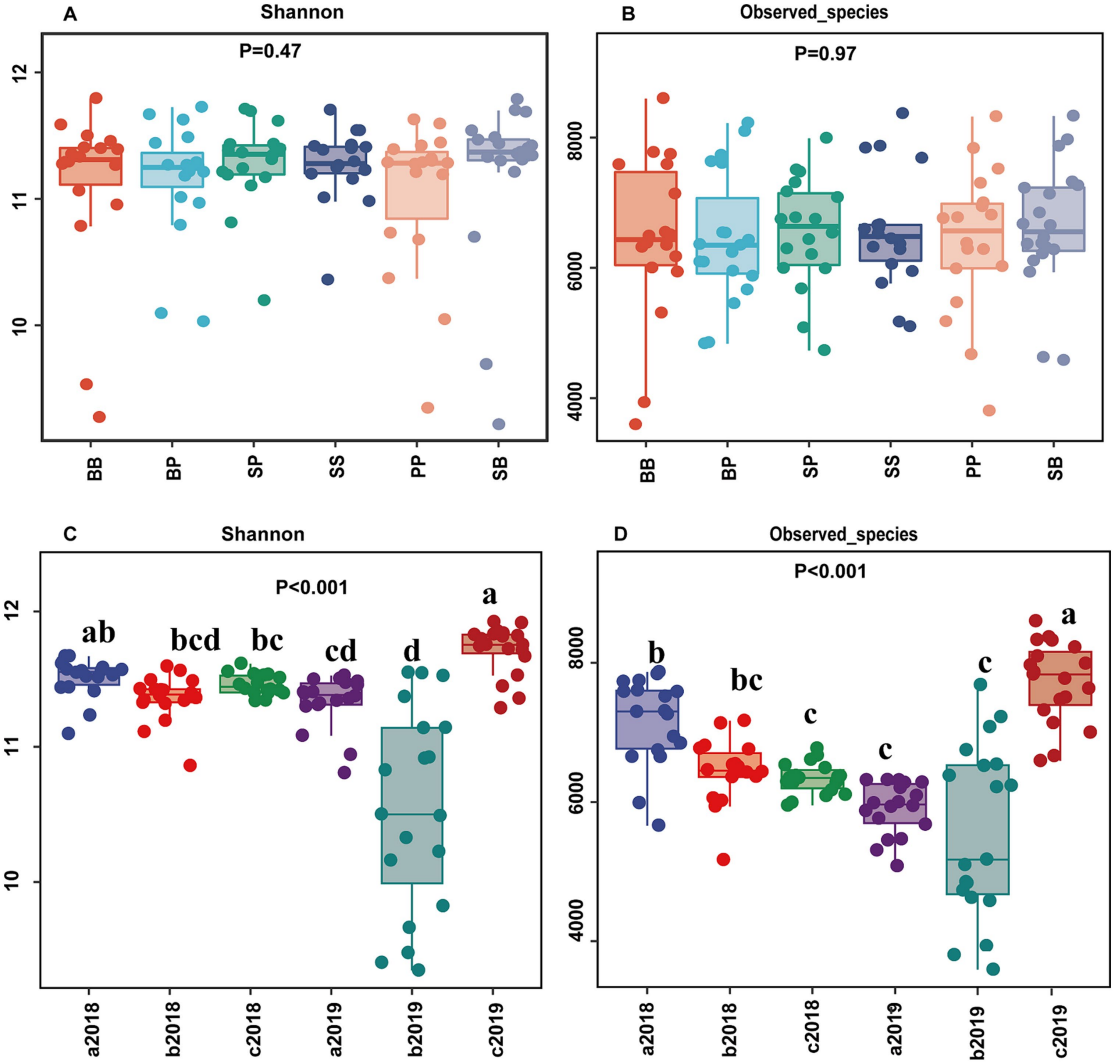
Alpha diversity of soil bacterial community (Shannon and observed species) across plant–plant interactions **(A,B)** and across different fertilizer treatments with time **(C,D)**. Different letters above the column indicate significant difference in **(C,D)** (*p* < 0.05) between treatments. a2018, b2018, and c2018 represent the treatment in control, inorganic fertilizer, and organic fertilizer in the year 2018, respectively; a2019, b2019, and c2019 represent the treatment in control, inorganic fertilizer, and organic fertilizer in the year 2019, respectively; The capital letter B, S, and P represent plant broadleaf species *Betula albosinensis*, shrub species *Salix oritrepha*, and conifer species *Picea asperata*, respectively; BB, SS, and PP refer to intraspecific plant–plant interactions, while SB, SP, and BP refer to interspecific plant–plant interactions.

The soil bacterial alpha diversity including Observed species (OTUs) and Shannon index, was significantly affected by both sample time and fertilization (*p* < 0.001) (Figure 2, Supplementary Table S2). However, neither plant–plant interaction nor the combined interactions of plant and fertilization had a significant influence on these diversity (*p* > 0.05). Regardless of the plant–plant interaction, the Observed species initially decreased in the organic fertilizer treatments compared to control but subsequently increased in the second year. The Shannon index and Observed species in inorganic treatment were lower than control but not significantly different between them. These diversities decreased with time in both control and inorganic fertilizer treatment while they increased with time in organic fertilizer treatments (Figure 2).

The PCoA showed the soil bacterial community was distinct across different fertilization treatment both in 2018 yr. and 2019 yr. (Supplementary Figure S1). The significant difference of beta diversity was detected by PERMNOVA test and the result showed that the soil bacterial community was significantly affected by both sample time (*F* = 9.50, *p* = 0.001) and fertilization (*F* = 22.18, *p* = 0.001), but was not affected by the plant–plant interactions (*F* = 1.07, *p* = 0.33) (Table 2).

**Table 2:** PERMANOVA tests for the effects of sample time, fertilization and plant–plant interaction on the soil bacterial community composition based on weighted UniFrac distances at OTU level.

Effects	*F*-value	*p*
Time (2018 vs. 2019)	9.50	0.001
Fertilization	22.18	0.001
CK vs. Inorganic	20.49	0.001
CK vs. Organic	10.12	0.001
Inorganic vs. Organic	29.79	0.001
Time × Fertilizer	18.60	0.001
Plant–plant interactions	1.07	0.33

### 3.4 Taxonomic composition of soil bacterial community

**Figure 3:**
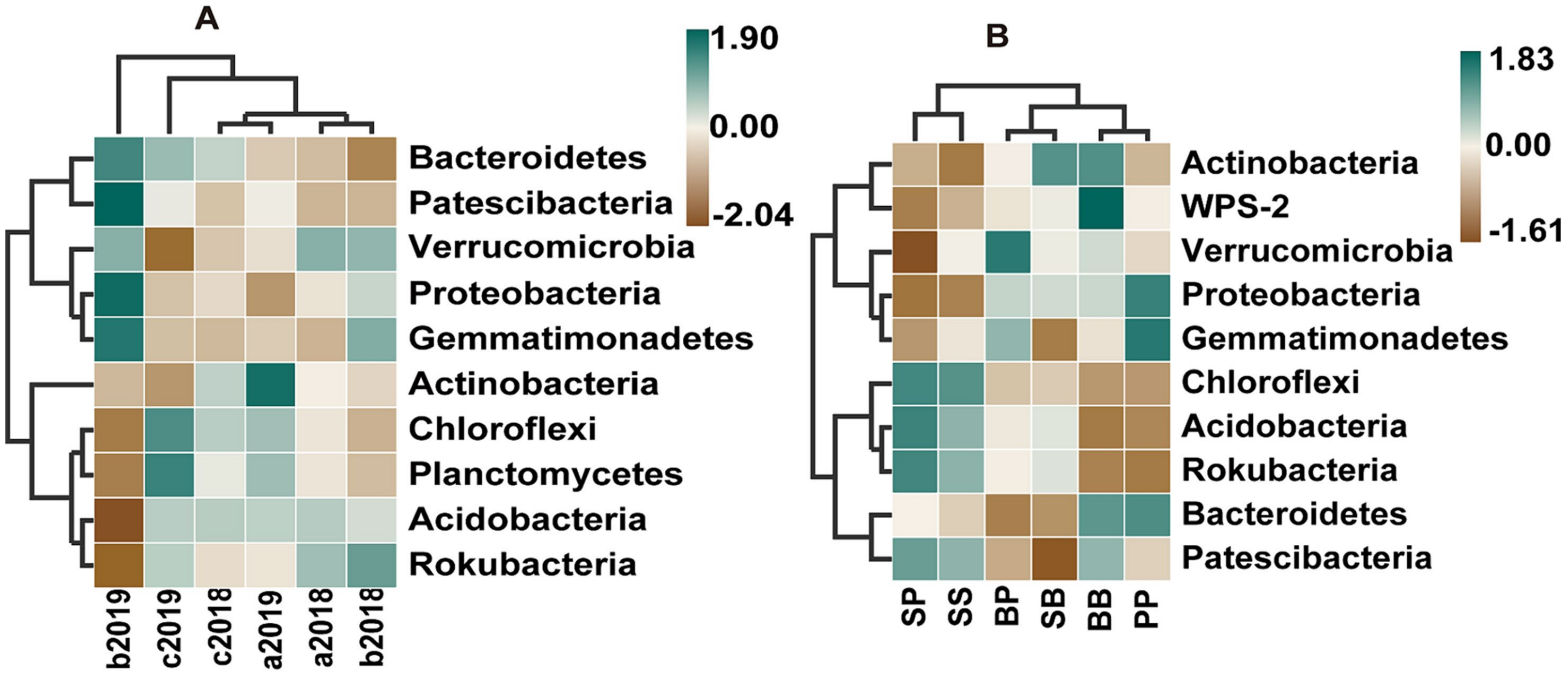
Heatmaps indicating differences in the relative abundances of soil bacterial community at phylum level (top 10) across different fertilizer treatments with time **(A)** and between plant–plant interactions under Inorganic fertilizer treatment in the 2019 yr. **(B)**. a2018, b2018, and c2018 represent the treatment in control, inorganic fertilizer, and organic fertilizer in the year 2018, respectively; a2019, b2019, and c2019 represent the treatment in control, inorganic fertilizer, and organic fertilizer in the year 2019, respectively; The capital letter B, S, and P represent plant broadleaf species *Betula albosinensis*, shrub species *Salix oritrepha*, and conifer species *Picea asperata*, respectively; BB, SS, and PP refer to intraspecific plant–plant interactions, while SB, SP, and BP refer to interspecific plant–plant interactions.

The soil bacterial communities were predominantly composed of *Proteobacteria*, *Acidobacteria*, *Actinobacteria*, *Chloroflexi*, *Verrucomicrobia*, and *Bacteroidetes* at the phylum level, accounting for 90% of total bacterial abundance (Supplementary Figure S2). Different fertilizers had their specific influence on the bacterial compositions, and these influences varied with sample time (Supplementary Figure S3). Irrespective of the plant–plant interactions, inorganic fertilizer increased the relative abundance of *Proteobacteria*, *Patescibacteria*, *Bacteroidetes* and *Gemmatimonadetes* and decreased the relative abundance of *Acidobacteria*, *Actinobacteria*, *Chloroflexi*, *Rokubacteria* and *Planctomycetes* in the second year compared to control. Whereas, organic fertilizer enhanced the relative abundance of *Chloroflexi*, *Planctomycetes* and *Bacteroidetes* across both years (2018 and 2019), while it decreased the relative abundance of *Actinobacteria* and *Verrucomicrobia* in the second year compared to control (Figure 3, Supplementary Table S3).

The LEfSe analysis indicated that, there were no statistically significant variations in the abundance of any taxa across different plant species or interspecific interactions. However, the total relative abundances of bacteria at phylum level were significantly affected by plant–plant interactions under inorganic fertilizer treatment in 2019 yr. (Figure 3 and Supplementary Table S3). In singular monocultures pots, while the *Proteobacteria* in the SS soil were less abundant than in BB and PP soil, the *Acidobacteria*, *Chloroflexi* and *Rokubacteria* was on the contrary under inorganic fertilizer treatment in the second year. In the mixed cultures pots, while the relative abundance of *Proteobacteria* in PP soil were more than in SP soil, the relative abundance of *Acidobacteria*, *Chloroflexi* and *Rokubacteria* in PP soil were less than in SP soil, and there was no difference between SS and SP under inorganic fertilizer treatment in the second year.

### 3.5 Environmental factors affected the soil bacterial community

**Figure 4:**
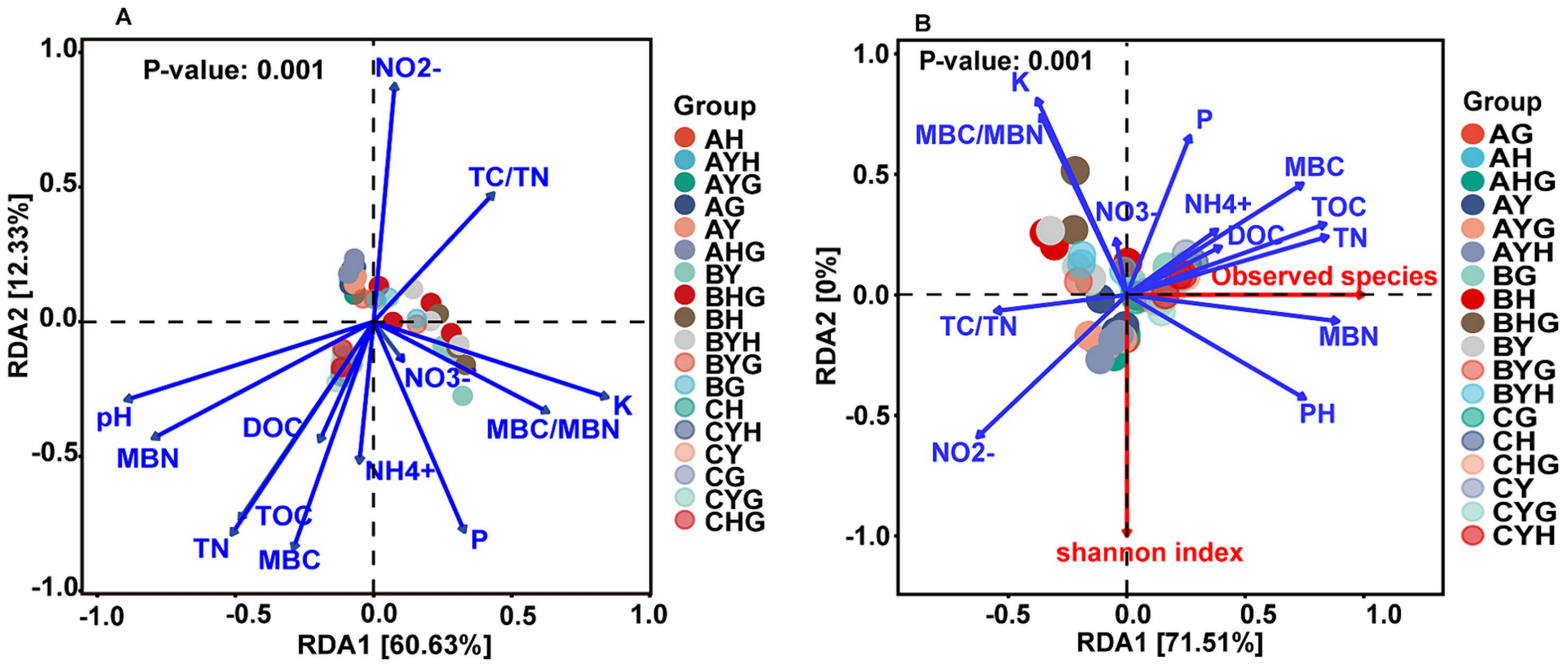
The effects of environmental factors on the bacterial communities **(A)** and alpha index **(B)** analyzed by redundancy analysis (RDA). DOC, dissolve organic carbon; TOC, total soil organic carbon; TN, total soil nitrogen; P, available phosphorus; K, available potassium; MBC, microbial biomass carbon; MBN, microbial biomass nitrogen.

The results of RDA analysis indicated that soil properties significantly affected the alpha diversity and compositions of the soil bacterial community (Figure 4). Among these soil properties, soil pH, TOC, TN, MBC, MBN were the most important factors influencing them. Besides, nutrients such as available K, available P, and NO_2_^−^ also affected the bacterial communities (Supplementary Table S4).

### 3.6 Factors affecting plant growth

**Figure 5:**
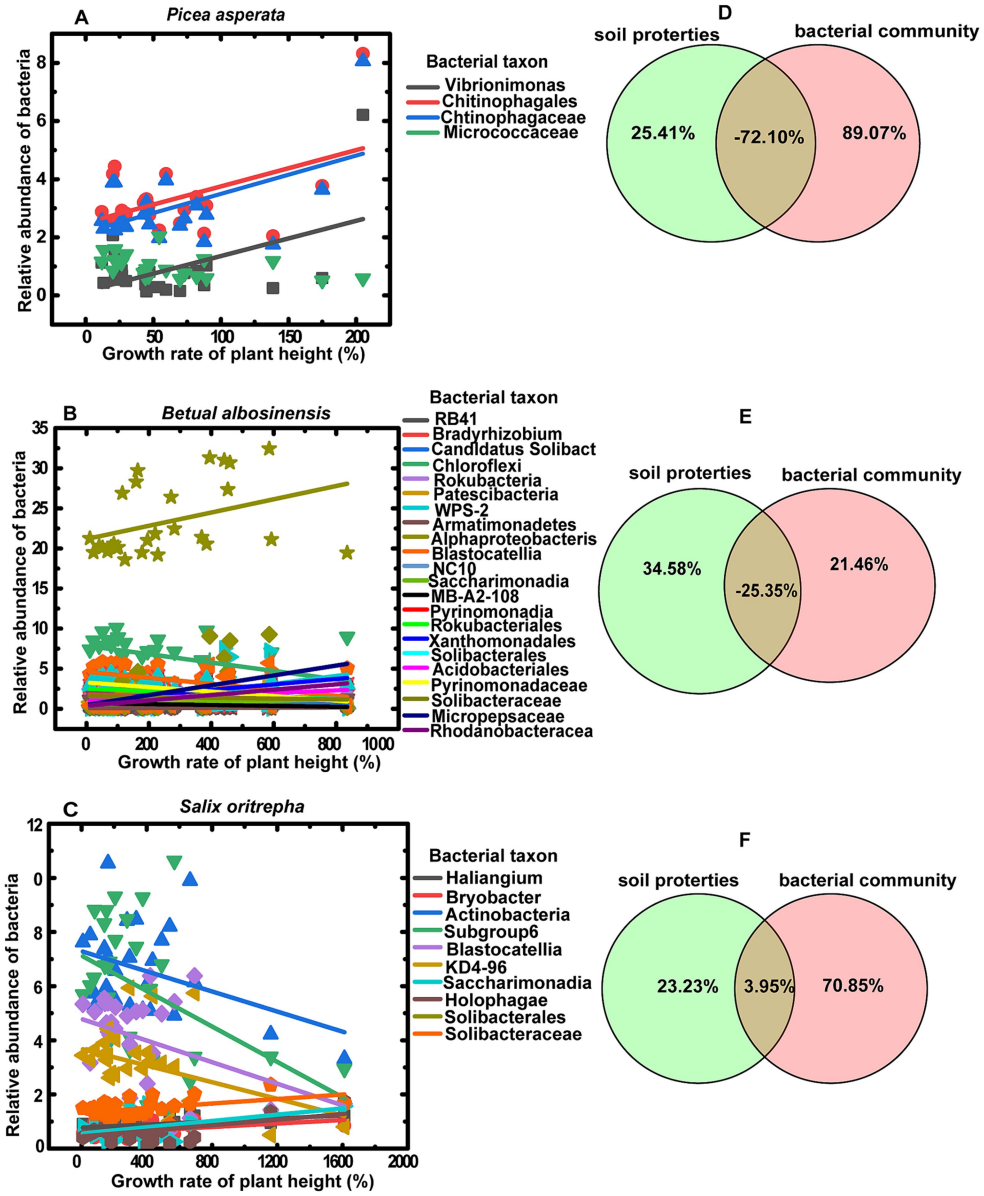
Linear regression of the relative abundances of bacteria taxa with growth rate of plant height **(A–C)** and variation partitioning analysis (VPN) depicted the shared and independent effects of soil properties and bacterial community on the growth rate of plant height for *Picea asperata* **(A,D)**, *Betula albosinensis* **(B,E)** and *Salix oritrepha* **(C,F)**. Only the regression lines significantly at least at *p* < 0.05 are presented.

The growth rate of conifer seedling (*P.asperata*) height exhibited a positive correlation with the relative abundances of bacteria genus *Vibrionimonas* (r = 0.49, *p* = 0.017), bacteria order *Chitinophagales* (r = 0.49, *p* = 0.016) and bacteria family *Chitinophagaceae* (r = 0.52, *p* = 0.010), but a negative correlation with bacteria family Micrococcaceae (r = −0.45, *p* = 0.032) (Figure 5A). And variation partitioning analysis indicated that growth rate of *P.asperata* was independently explained by bacterial communities (89.07%) rather than soil properties (25.41%) (Figure 5D). Similarly, the bacterial communities could independently explain the growth rate of shrub seedling (*S.oritrepha*) height greater compared to soil properties (70.85 and 23.23%, respectively) (Figure 5E). Whereas the growth rate of broadleaf seedling (*B.albosinensis*) height was affected by more bacterial taxa (Figure 5C), but the bacterial communities only independently explained its growth about 21.46% (Figure 5F).

## 4 Discussion

### 4.1 Plant interaction performance

Due to the rapid growth and extensive root systems of shrub seedling (*S.oritrepha*) when mixed with other plants, it was not feasible to separate the root. Therefore, our analysis of the plant–plant interaction was based on growth rate of plant height, as aboveground biomass is likely influenced by initial difference caused by the size of seedling. Although some plants modify the balance between aboveground and belowground allocation in response to competition, the majority of these adjustments are constrained, meaning that they are mainly influenced by competition on plant size (Mülle et al., 2000). Thus, growth rate of plant height will be a more suitable indictor for inflecting the influence of plant–plant interactions. We initially assumed that conifer species would be a superior competitor when grown in mixture with other species, according to the rule of secondary forest succession, which indicates that mid-successional plants are eventually replaced by late-successional plants. In this study, *B.albosinensis* and *S.oritrepha* grew faster than *P.asperata*. The interspecific interaction only affected the plant growth in the organic fertilizer treatment but not in the control and inorganic fertilizer treatment. Numerous studies have exhibited that plants tend to suffer competitive interactions from their neighbors (Guo et al., 2019; Cavalieri et al., 2020). However, in this study, the application of organic fertilizer resulted in the facilitation of *P.asperata* growth by *B.albosinensis* and *S.oritrepha*. *S.oritreph* promoted the growth of *B.albosinensis*, while *P.asperata* also promoted growth of *S.oritreph*. These findings exceed our initial hypothesis and further suggest that taking certain manual measures could make the mid-successional species promoting the growth of later successional species, thereby facilitating secondary forest succession. The positive influence of inorganic fertilizer on plant growth and the plant–plant interactions were widely studied (Yu et al., 2019; Cavalieri et al., 2020; Ma et al., 2022). In this study, Inorganic fertilizer only enhanced the growth rate of *P.asperata* and *B.albosinensis* in PB mixture and *S.oritrepha* in SB mixture and SS monocultures but not for *P.asperata* and *B.albosinensis* in singular monoculture compared to control. These results suggested that the effect of inorganic fertilizer on plant growth depends on the composition of the plants as different plants have different nutrients acquisition and different needs.

### 4.2 Responses of diversity and composition of bacterial communities to different fertilizers

Changes in soil bacterial diversity and composition reflect the soil quality (Bending et al., 2004), as well as the maintaining integrity and stability of soil function (Dietrich et al., 2017). A meta-analysis investigating the effects of organic and mineral fertilizers on soil microbial diversity revealed no significant variation in the bacterial alpha diversity between NPK fertilizer treatment and control, but a 2.9% increase was observed in organic fertilizer treatment compared to control (Bebber and Richards, 2022). Similarly, Ma et al. (2022) found no significant difference in bacterial diversity in paddy soil between inorganic fertilizer and control; however, higher bacterial diversity was observed with organic fertilizer application compared to control. In consistent with these findings, our results indicate that while inorganic fertilizer (NPK) did not have significant effect on the soil bacterial alpha diversity, organic fertilizer increased it during the second year. The abundance and diversity of microbial communities are found to be closely associated with the amount of soil microbial biomass. Organic fertilizer contains not only NPK but also undigested plant matter (lignin, cellulose, hemicellulose), lipids, carbohydrates and essential nutrients (Cu, Fe, Zn, Mg), which may contribute to a higher microbial diversity according to species-energy hypothesis since organic fertilizer increase soil microbial biomass more effectively than NPK fertilizer do (Clarke and Gaston, 2006). In this study, organic fertilizer enhanced the soil organic carbon and microbial biomass but inorganic fertilizer only enhanced the soil microbial biomass and did not significantly affect the soil organic carbon. The observed increase in soil microbial biomass was more pronounced with organic fertilizer treatment compared to inorganic fertilizer treatment, which is consistent with findings from previous studies (Zhang et al., 2012; Francioli et al., 2016; Kumar et al., 2017). Furthermore, the bacterial diversity was mainly mediated by the soil properties such as soil pH, TOC, and N content, and pH was considered as the most important moderator to control the diversity (Zhou et al., 2020; Yu et al., 2021). Bebber and Richards (2022) found that the N and NPK fertilizer decreased soil pH, but manure fertilizer had a buffering effect on pH. This buffering effect, along with the presence of nutrient and organic content, is considered to be the primary advantage for microbial biodiversity. In this study, we observed a positive correlation between bacterial alpha-diversity (Observed species and Shannon index) and soil pH, TN, NH_4_^+^-N, MBC, MBN, K; conversely, there was a negative correlation between bacterial alpha-diversity and soil NO_2_^−^-N, and TC/TN (Supplementary Table S4). The application of inorganic fertilizer (NPK) increased soil MBC, available K and NH_4_^+^-N concentration, but reduced soil MBN, pH, and finally slightly but not significantly decreased the soil bacterial diversity. On the other hand, the organic fertilizer resulted in an increase in soil properties such as pH, TOC, TN, NH_4_^+^-N, MBC, MBN and available K, leading to an increase in soil bacterial diversity during the second year. These findings suggest that the impacts of different fertilizers on soil bacterial diversity depends on the integrated effects of those combined soil factors affected by them.

The addition of Nitrogen (N), phosphorus (P) and potassium (K) fertilizer directly influences bacterial composition by increasing the availability of N, P and K (Guo et al., 2019; Cheng et al., 2022). In this study, both inorganic fertilizer and organic fertilizer enhanced the concentrations of soil NH_4_^+^, available P and available K. The PCoA and PERMANOVA analyses showed significant effects on the bacterial composition resulting from the addition of inorganic and organic fertilizer (Table 2). *Actinobacteria, Proteobacteria, Bacteroidetes*, *Acidobacteria, Chloroflexi* and *Verrucomicrobia* were found to be the most abundant bacterial groups accounting for approximately 90% of bacterial abundance. These six bacterial groups can be classified into copitroph *r*-strategists and oligotroph *K*-strategists according to their life history strategies. *Actinobacteria, Proteobacteria, Bacteroidetes* are generally regarded as r-strategists in the copiotrophic group, and *Acidobacteria, Chloroflexi* and *Verrucomicrobia* are regarded as K-strategists in the oligotrophic group (Xu et al., 2020; Ma et al., 2022). The addition of organic fertilizer enhanced the relative abundances of *r*-strategists in the copiotrophic group (*Actinobacteria*, *Bacteroidetes* and *Proteobacteria*), whereas inorganic fertilizer increased those of K-strategists in the oligotrophic group (*Acidobacteria, Chloroflexi* and *Verrucomicrobia*) (Xun et al., 2016). Additionally, Ma et al. (2022) found that the application of organic fertilizer increased the abundance of *r*-strategists in the copiotrophic group (*Actinobacteria* and *Proteobacteria*) but decreased those of K-strategists in the oligotrophic group (*Acidobacteria,* and *Verrucomicrobia*). Fierer et al. (2012) observed that an increase in soil N availability led to a higher abundance of copiotrophic bacteria taxa such as *Proteobacteria* and *Bacteroidetes*, while reducing the abundance of oligotrophic group *Acidobacteria*. Consistently, our study observed that compared to control, both organic and inorganic fertilizer resulted in an increased relative abundance of copiotrophic group *Bacteroidetes* and *Proteobacteria* (*Alphaproteobacteria* and *Dongiale*), whereas the oligotrophic group *Acidobacteria* and *Verrucomicrobia* abundances were decreased by organic fertilizer. In this study, some bacteria belonging to *Acidobacteria* like *Holophagae* and *Thermoanaerobaculia* were reduced by inorganic fertilizer (Xun et al., 2016), whereas bacterial phylum *Acidobacteria* were enhanced (Fierer et al., 2012). Additionally, *Chloroflexi* was regarded as oligotrophic group and its relative abundance was lower in the fertilized soils than control soil (Xu et al., 2020) but increased by inorganic fertilizer (Xun et al., 2016). However, in this study, organic fertilizer increased the relative abundance of *Chloroflexi*, whereas inorganic fertilizer decreased it compared to control during the second year. This observation can be explained by the positive correlation between the relative abundance of *Chloroflexi* and soil pH (Deng et al., 2018). *Actinobacteria*, known as a copiotrophic group, played an important role in the degradation of soil organic matters and exhibited higher relative abundance both in the organic and inorganic fertilizer soils (Francioli et al., 2016; Xu et al., 2020; Ma et al., 2022). We observed a decrease in the relative abundances of bacteria phylum *Actinobacteria* with the addition of both inorganic and organic fertilizer, but also observed increase in *Frankiales* and *Micromonosporales* belonging to *Actinobacteria* in the inorganic fertilizer treatment. Moreover, the inorganic fertilizer increased the total relative abundances of the r-strategists in copiotrophic group (*Actinobacteria*, *Bacteroidetes* and *Proteobacteria*) by approximately 3.53% and decreased the K-strategists in oligotrophic group (*Acidobacteria, Chloroflexi* and *Verrucomicrobia*) by 5.45%. Conversely, although organic fertilizer adjusted the composition of bacterial community only within its respective group, it had no significant effect on the total relative abundances of either copiotrophic or oligotrophic groups compared to control in this study. Notably, discussing bacterial communities’ responses to different fertilizers at the genus or order level may be more precise and effective in drawing conclusions than at the phylum level. For instance, certain findings in this study were inconsistent with previous studies conducted in agriculture soil or grassland, particularly at the bacterial phylum level. This underscores the significant role of vegetation in releasing root exudates, while also potentially being influenced by factors operating at the phylum level rather than genus or order level.

### 4.3 Fertilizer affect the response of the bacterial composition to plant–plant interactions

There are many studies showed that different plants have specific effects on rhizosphere soil physicochemical properties through root exudates and nutrient mineralization (Guo et al., 2019), and then the plant species significantly have great impact on the composition and diversity of soil bacterial communities both directly and indirectly (De Deyn et al., 2011; Chen et al., 2016; Wang et al., 2022; Zhang et al., 2024). Also numerous research showed that interspecific plant–plant interaction influenced soil microbial communities due to competition for nutrients or different plants release different exudates which will affect the soil bacterial community (Haichar et al., 2008; Knelman et al., 2018; Cavalieri et al., 2020). In this study, we observed significant effects of plant species across various stages of secondary succession in forest restoration as well as their inter-specific interactions on soil properties including the concentrations of DOC, NH_4_^+^, AP, AK, MBC, MBN, particularly under fertilization treatments. These factors exhibited strong correlations with the diversity and composition of soil bacterial communities (Figure 4), suggesting that the plant species would have a significant impact on bacterial communities. However, neither the plant species in monocultures nor the plant–plant interactions in mixture had significant influence on the diversity and composition of soil bacterial community in the control. This result suggests that the rhizosphere soil bacterial communities diversity remains stable across secondary forest succession and factors other than plant diversity may be driving variations in soil communities. Pivato et al. (2017) found that the total bacterial abundance in the rhizosphere of inter-specific plant–plant mixtures was significantly greater than that of either individual plant species in a monoculture at low N levels, but this difference disappeared when the N levels were high in agroecosystems. In this study, the responses of bacterial community abundances in the rhizosphere of late-successional species *P.asperata* (P) to inorganic fertilizer was found to be the most sensitive, while the mid-successional species shrub *S.oritrepha* (S) exhibited the least sensitivity. This resulted in significant difference in the relative abundances of *Proteobacteria*, *Acidobacteria*, *Chloroflexi* and *Rokubacteria* between in the rhizosphere of SS and PP or BB monocultures, as well as between in the rhizosphere of PP and SP mixture under inorganic fertilizer treatment during the second year. This result further suggests that different successional species had varying response to fertilizer application.

When two plant species coexist, how does interspecific interaction select their bacterial community structure? Hortal et al. (2017) reported that when two plant species were grown together in mixed cultures, the composition of rhizoshpere bacterial community resembled that of the superior competitor in monocultures. We initially assumed that conifer species is superior competitor and their rhizoshpere soil bacterial community would resemble that of their monocultures soil when grown with other species in mixture. However, contrary to this expectation, our study indicates that the bacterial communities in SP soil are more similar to SS soil rather than PP soil, and similarly for SB soil compared to BB soil under inorganic fertilizer treatment (Figure 3). Interestingly, a similar pattern was observed between P and B interaction. This discrepancy may be attributed to differences in plant growth rate and root development. Our former results of plant growth indicated that the seedling growth and root development of shrubs are comparatively faster than broadleaf seedlings, while confer seedlings exhibit the slowest growth. Consequently, shrub roots produce a greater quantity of root exudates compared to broadleaf or conifer roots that significantly influence the composition and diversity of soil bacterial communities. Furthermore, while the organic fertilizer had similar impacts on the soil chemical properties and microbial biomass as the inorganic fertilizer, it also promoted plant growth and affected the interspecific interaction. However, it did not alter the effects of plant species or plant–plant interaction on the rhizosphere bacterial communities. These results imply that organic fertilizer not only improves soil nutrient availability but also maintains soil health and promotes the succession of plant communities compared to inorganic fertilizer due to its potential influence on nutrients cycling and organic matter decomposition, and plants disease susceptibility mediated by changes in bacterial community composition. Therefore, it is important to consider the potential effects of different fertilizer management practices on soil bacterial communities when managing soil resources for forest recovery.

### 4.4 Bacterial communities contributed to plant growth

It is widely acknowledged that soil properties and bacterial communities exert significant influences on plant growth (Kardol et al., 2006), suggesting that plant–plant interactions and fertilizers may affect plant growth by regulating soil properties and bacterial communities. In this study, although both the plant–plant interactions and fertilizers influenced the soil properties and the soil bacterial communities also were mediated by soil properties (Figure 4), the variance partitioning analysis (VPA) results revealed that the growth rates of coniferous seedling *P.asperata* and shrub seedling *S.oritrpha* were predominantly explained by bacterial communities (89.07 and 70.85%, respectively), rather than soil properties alone (25.41 and 23.23%, respectively) (Figures 5D,F). Moreover, the growth rate of broadleaf seedling *B.albosinensis* was found to be more strongly affected by soil properties compared to bacterial communities (Figure 5C). Nevertheless, certain bacterial taxa such as *Patescibacteria*, *WPS-2*, *Armatimonadetes*, *Alphaproteobacteria*, etc., exhibited significantly positive correlations with plant growth rate, whereas others including *RB41*, *Bradyrhizobium*, *Chloroflexi*, *Rokubacteria*, etc., showed negative correlations (Supplementary Table S5). Ultimately, the effects of different bacterial taxa on plant growth rate counterbalanced each other. These findings underscore the substantial impact of soil bacterial communities on plant growth. In line with our results, numerous studies investigating the effect of plant–soil feedback have consistently demonstrated a strong association between plant performances and soil biota rather than abiotic conditions (Kardol et al., 2006). Plant growth-promoting bacteria (PGPB) play a crucial role in enhancing plant growth and crop productivity in agroecosystems. Extensive research has been conducted on various plant growth promoting communities, such as *Actinomycetota*, *Acidobacteria*, and *Chloroflexi*, which have been proven to drive rhizosphere ecology and nutrient cycling. These communities promote plant growth and fitness through mechanisms like phosphate solubilization, secondary metabolite production, and antimicrobial synthesis (Kaushik et al., 2021). In this study, plants across different successional stages of secondary forest had specific bacterial communities for promoting their growth (Figures 5A–C). This finding offer valuable perspectives on the potential regulatory mechanisms of microbial ecology during the progression of plant community succession, underscoring the significance of microbial communities in driving forest succession. This understanding will aid in the development of more effective strategies for optimizing forest restoration practices. For example, promoting beneficial rhizosphere bacteria can improve nutrient cycling and facilitate the establishment of diverse native plant communities in degraded forests, ultimately contributing to overall ecosystem resilience in managed forests. Therefore, understanding the responses of bacterial communities to plant–plant interaction and different types of fertilizers can help us develop sustainable strategies for managing forests and maximizing their ecological benefits. Overall, this study highlight the critical role of bacteria in shaping forest ecosystems during succession. Further investigations are needed to unravel additional mechanisms employed by these bacteria as well as explore their potential application.

## 5 Conclusion

Our results indicated that both the plant species and interspecific interactions, as well as fertilizer application, significantly affected soil properties. However, the responses of plant growth rate and bacterial community to plant–plant interactions and fertilizer exceeded our initial expectations. We initially hypothesized that different dominant plant species across various successional stages would exhibit distinct rhizosphere microbial communities, with the rhizoshpere bacterial communities resembling those of superior competitor later successional conifer seedling in monocultures when grown with other species in mixture. In this study, the growth rate of plant height varied with plant species, but the bacterial communities of different plants remained stable across successional stages in control. The application of inorganic fertilizer has the potential to modify the composition of bacterial communities and influences plant–plant interactions but did not significantly alter the interspecific interaction on plant growth. And contrary to our initial hypothesis, bacterial communities in SP soil are more similar to SS soil rather than PP soil, and similarly for SB soil compared to BB soil under inorganic fertilizer treatment. Whereas organic fertilizer resulted in an increase in bacterial diversity, changes in bacterial communities, as well as facilitating later successional species’ growth by the mid-successional specie, ultimately facilitating the secondary forest succession. Moreover, the correlations of plant growth with the relative abundances of certain bacteria taxa, along with VPN results, emphasize that plants at different successional stages of secondary forest harbor specific bacterial communities to affect their growth, further suggesting the significance of bacterial communities for plant community’s succession. Overall, these findings from our pot experiment underscore the importance of considering both plant–plant interactions and diverse fertilizer types in forest restoration efforts and provide valuable insights into optimizing agronomic practices for secondary forest succession while simultaneously safeguarding soil health and biodiversity, thereby mitigating any adverse effects of fertilizer management practices on soil microbial communities. These findings will contribute to the provision of technological support for the restoration of degraded forest. However, further research is still required conducted in field to provide more practical support for forest restoration.

## Data Availability

Sequence data have been deposited in the NCBI Sequence Read Archive (SRA) under BioProject ID PRJNA1073250.

## Footnotes

^1^ https://docs.qiime2.org/2019.4/tutorials

^2^ https://www.genescloud.cn
