# Supplementary material for: Different responses of soil bacterial community to plant–plant interactions under organic–inorganic fertilizers affect seedling establishment during subalpine forest succession
Source: Front Microbiol. 2024 Oct 1;15:1466668. doi: 10.3389/fmicb.2024.1466668 (PMC11473419; doi:10.3389/fmicb.2024.1466668)

Supplementary Material

Different responses of soil bacterial community to plant-plant interactions under organic-inorganic fertilizers affect seedling establishment during subalpine forest succession

Dandan Li[1], Yongping Kou[1], Jin Liang[1], Wenqiang Zhao[1], Dongdong Chen[1], Qing Liu[1]*

[1]CAS Key Laboratory of Mountain Ecological Restoration and Bioresource Utilization & Ecological Restoration Biodiversity Conservation Key Laboratory of Sichuan Province, Chengdu Institute of Biology, Chinese Academy of Sciences, Chengdu, China

*** Correspondence:**
Corresponding Author: Qiu Liu
E-mail: liuqing@cib.ac.cn

Table S1 Soil properties varied with plant-plant interactions under different fertilizer treatments (Mean ± SE).

	Plant-plant	pH	DOC(mg/kg)	TOC(g/kg)	TN(g/kg)	TC/TN	NO_3^-(mg/kg)	NH_4^+(mg/kg)	NO_2^-(mg/kg)	AP(mg/kg)	AK(mg/kg)	MBC(mg/kg)	MBN(mg/kg)	MBC/MBN
Control	SS	6.55±0.02	118.67±18.44	51.69±0.40	3.97±0.06	13.02±0.24	4.73±2.46	5.42±0.10	0.19±0.00	5.35±0.31	78.33±2.19	253.01±18.14	84.67±5.57	3.03±0.37
	BB	6.58±0.04	108.44±5.81	52.29±0.20	3.93±0.02	13.31±0.09	4.44±1.11	6.86±0.55	0.24±0.01	5.84±0.28	87.33±5.04	271.23±11.59	78.46±3.57	3.47±0.21
	SB	6.53±0.02	133.13±9.12	52.00±1.11	3.84±0.03	13.55±0.33	2.46±0.75	6.12±1.31	0.17±0.00	4.72±0.15	113.67±42.01	259.62±2.20	86.60±2.97	3.00±0.08
	PP	6.55±0.01	83.14±1.81	51.42±1.34	3.92±0.02	13.11±0.28	5.70±0.88	5.32±0.43	0.19±0.01	7.36±0.35	86.00±4.36	286.01±4.01	72.56±1.79	3.94±0.04
	SP	6.69±0.04	146.99±15.97	52.13±0.94	3.91±0.02	13.35±0.19	1.67±0.15	5.53±0.39	0.19±0.01	5.96±0.31	66.33±2.19	337.24±35.48	96.35±11.98	3.52±0.14
	BP	6.61±0.01	104.22±8.69	52.17±0.38	3.91±0.03	13.34±0.15	5.25±1.33	5.10±0.20	0.22±0.01	6.22±0.61	118.00±25.00	321.85±29.38	52.83±11.56	6.64±1.27
Inorganic fertilization	SS	5.74±0.12	142.17±12.10	52.72±1.03	3.83±0.06	13.78±0.11	1.51±0.16	6.15±0.41	0.07±0.00	53.53±9.34	463.33±40.55	586.88±37.36	65.72±12.52	9.51±1.54
	BB	5.98±0.02	128.31±13.81	52.06±1.84	4.09±0.06	12.72±0.47	3.47±1.22	7.55±0.68	0.10±0.01	39.73±9.08	566.67±31.80	390.12±21.05	38.75±4.09	10.38±1.48
	SB	5.91±0.06	134.34±8.69	53.98±2.15	4.02±0.07	13.42±0.30	9.73±7.63	6.21±0.94	0.15±0.00	28.41±10.82	496.67±49.78	455.94±20.97	70.93±2.69	6.46±0.48
	PP	5.82±0.09	103.62±9.64	52.41±1.64	3.77±0.06	13.89±0.40	4.67±1.68	7.22±0.30	0.15±0.00	85.72±7.93	486.67±38.44	391.84±24.67	33.80±3.10	11.89±1.79
	SP	5.89±0.09	140.96±14.04	52.73±1.22	3.92±0.07	13.43±0.29	1.84±0.23	7.20±0.52	0.10±0.01	30.55±7.13	486.67±85.70	574.63±14.68	76.61±10.66	7.79±1.04
	BP	5.83±0.00	137.35±6.26	52.44±0.94	4.00±0.03	13.10±0.27	2.87±0.43	6.84±0.17	0.10±0.01	47.31±22.78	636.67±96.84	590.00±93.11	46.61±4.70	12.54±1.14
Organic fertilization	SS	6.97±0.04	204.82±12.33	61.67±2.87	5.38±0.29	11.49±0.23	2.28±0.14	11.26±0.10	0.03±0.01	39.95±3.84	185.33±4.37	928.38±89.29	151.65±7.67	6.10±0.35
	BB	6.90±0.09	153.01±19.02	64.31±2.90	5.29±0.17	12.21±0.93	7.30±0.97	6.90±0.75	0.07±0.00	49.44±6.79	245.00±18.23	802.51±37.81	123.08±4.60	6.52±0.23
	SB	6.97±0.03	166.57±4.25	65.23±2.66	5.35±0.23	12.20±0.08	3.15±0.30	8.53±0.22	0.02±0.00	46.90±3.74	209.67±16.29	922.77±42.62	146.00±6.13	6.32±0.10
	PP	7.01±0.04	119.27±8.91	65.29±0.84	5.19±0.02	12.59±0.18	5.50±2.05	7.36±0.40	0.04±0.00	33.66±2.26	159.33±5.24	771.63±33.72	113.97±7.11	6.79±0.16
	SP	6.99±0.01	139.76±4.93	61.39±2.06	5.22±0.07	11.75±0.24	3.67±0.28	8.58±1.11	0.03±0.02	49.41±0.81	213.00±9.64	742.30±44.64	140.12±18.02	5.48±0.76
	BP	7.10±0.01	157.23±3.61	72.37±4.07	5.99±0.32	12.09±0.20	5.29±0.92	5.83±0.50	0.05±0.01	65.44±6.67	257.67±19.19	832.52±13.73	126.33±2.88	6.59±0.06

DOC: dissolve organic carbon, TOC: total soil organic carbon, TN: total soil nitrogen, AP: available phosphorus, AK: available potassium, MBC: microbial biomass carbon, MBN: microbial biomass nitrogen. The capital letter B, S, and P represent plant broadleaf specie *Betula albosinensis*, shrub specie *Salix oritrepha*, and conifer specie *Picea asperata*, respectively; BB, SS, and PP refer to intraspecific plant-plant interactions, while SB, SP, and BP refer to interspecific plant-plant interactions.

Table S2 Factorial ANOVA analysis of the effects of sample time, fertilization and plant-plant interactions on alpha diversity of soil bacterial communities.

		Chao1	Observed_species	Pielou_e	shannon	simpson
**Year**	F	**4.47***	**4.33***	**20.61*****	**15.51*****	**5.50***
	P	0.04	0.04	0.00	0.00	0.02
**Fertilization**	F	**13.59*****	**22.48*****	**43.17*****	**36.63*****	**7.27**
	P	0.00	0.00	0.00	0.00	0.00
**Plant**	F	0.16	0.25	2.29	1.38	0.52
	P	0.98	0.94	0.05	0.24	0.76
**Y *F**	F	**43.74*****	**41.37*****	**29.04*****	**24.36*****	**5.11****
	P	0.00	0.00	0.00	0.00	0.01
**Y*P**	F	1.07	0.99	0.98	1.02	0.60
	P	0.39	0.43	0.43	0.41	0.70
**F*P**	F	1.07	1.31	1.23	1.29	0.73
	P	0.40	0.24	0.29	0.25	0.69
**Y*F*P**	F	1.21	1.23	1.12	1.11	0.84
	P	0.30	0.28	0.36	0.37	0.60

*, **, *** represent the significant difference <0.05, <0.01, <0.001, respectively.

Table S3 Factorial ANOVA analysis of the effects of sample time, fertilization and plant-plant interactions on the relative abundance of dominated soil bacterial communities in phylum (F-value).

Phylum	Year	Fertilization	Plant	Y * F	Y * P	F * P	Y * F * P
Proteobacteria	0.15	36.74***	4.42***	15.66***	3.23*	0.99	1.03
Acidobacteria	38.45***	53.96***	1.59	35.04***	2.93*	2.14*	1.61
Actinobacteria	0.01	4.47*	2.31	5.51**	0.48	0.85	1.43
Chloroflexi	0.87	37.16***	8.66***	10.55***	3.44**	3.29***	2.65**
Verrucomicrobia	1.40	6.12**	1.16	1.60	0.88	0.82	0.38
Bacteroidetes	10.28**	3.52*	1.67	5.95**	1.82	0.97	0.70
Gemmatimonadetes	7.64**	106.08***	0.95	2.10	0.91	0.86	1.41
Rokubacteria	44.58***	6.09**	2.09	48.25***	2.84*	3.14**	3.02**
Planctomycetes	6.85*	28.23***	1.12	7.30***	0.66	1.09	1.70
Patescibacteria	50.01***	12.09***	1.45	9.59***	2.06	1.77	1.41

*, **, *** represent the significant difference <0.05, <0.01, <0.001, respectively.

Table S4 the effects of environmental factors on the soil bacterial community based on Bray-Curtis and alpha index analyzed by the redundancy analysis (RDA).

	**Bacterial community**				**Alpha index**			
	RDA1	RDA2	r^2	P value	RDA1	RDA2	r^2	P value
NO_3^-	0.587	-0.810	0.025	0.545	-0.095	0.996	0.041	0.349
NH_4^+	-0.209	-0.978	0.234	0.001	0.786	0.619	0.175	0.014
NO_2^-	0.197	0.980	0.660	0.001	-0.712	-0.702	0.606	0.001
DOC	-0.483	-0.876	0.213	0.005	0.858	0.514	0.156	0.011
pH	-0.929	-0.370	0.822	0.001	0.885	-0.466	0.500	0.001
TOC	-0.602	-0.798	0.716	0.001	0.911	0.413	0.610	0.001
TN	-0.598	-0.802	0.821	0.001	0.932	0.362	0.594	0.001
P	0.331	-0.944	0.549	0.001	0.423	0.906	0.406	0.001
K	0.977	-0.211	0.647	0.001	-0.343	0.939	0.558	0.001
MBC	-0.416	-0.909	0.708	0.001	0.816	0.577	0.609	0.001
MBN	-0.863	-0.505	0.777	0.001	1.000	-0.026	0.570	0.001
MBC/MBN	0.922	-0.388	0.403	0.001	-0.362	0.932	0.476	0.001
TC/TN	0.698	0.716	0.389	0.001	-0.975	-0.220	0.233	0.002

Table S5 the coefficients of correlation between growth rates of plant height and bacterial taxa.

**Plant species**	**Bacterial taxon**	**Pearson's r**	**P**
**Picea asperata**	Vibrionimonas	0.492	0.017
	Chitinophagales	0.497	0.016
	Chitinophagaceae	0.523	0.01
	Micrococcaceae	-0.448	0.032
**Betual albosinensis**	RB41	-0.424	0.024
	Bradyrhizobium	-0.458	0.014
	Candidatus Solibacter	-0.426	0.024
	Chloroflexi	-0.407	0.031
	Rokubacteria	-0.428	0.023
	Patescibacteria	0.382	0.044
	WPS-2	0.433	0.021
	Armatimonadetes	0.434	0.021
	Alphaproteobacteria	0.376	0.048
	Blastocatellia (Subgroup 4)	-0.403	0.033
	NC10	-0.428	0.023
	Saccharimonadia	0.412	0.029
	MB-A2-108	-0.435	0.021
	Pyrinomonadales	-0.425	0.024
	Rokubacteriales	-0.441	0.019
	Xanthomonadales	0.401	0.034
	Solibacterales	-0.399	0.035
	Acidobacteriales	0.388	0.041
	Pyrinomonadaceae	-0.425	0.024

	Solibacteraceae (Subgroup 3)	-0.399	0.035
	Micropepsaceae	0.419	0.027
	Rhodanobacteraceae	0.421	0.026
**Salix oritrepha**	Haliangium	0.392	0.039
	Bryobacter	0.475	0.01
	Actinobacteria	-0.390	0.04
	Subgroup 6	-0.446	0.017
	Blastocatellia (Subgroup 4)	-0.413	0.029
	KD4-96	-0.383	0.044
	Saccharimonadia	0.463	0.013
	Holophagae	0.507	0.006
	Solibacterales	0.442	0.018
	Solibacteraceae (Subgroup 3)	0.442	0.018

Figure S1. The PCoA plots for soil bacterial community based on Weighted UniFrac distances in 2018yr (A) and 2019yr (B). The former letters A, B, and C represent the fertilization treatment in the control, inorganic fertilizer, and organic fertilizer respectively in the legend. While the later letter B, S, and P represent plant broadleaf specie *Betula albosinensis*, shrub specie *Salix oritrepha*, and conifer specie *Picea asperata*, respectively; BB, SS, and PP refer to intraspecific plant-plant interactions, while SB, SP, and BP refer to interspecific plant-plant interactions.

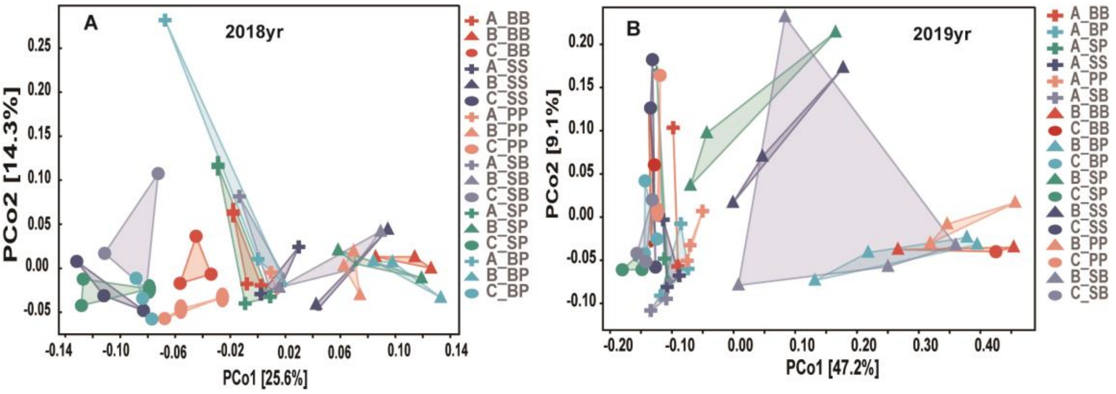

Figure S2. The relative abundance of soil bacterial community at phylum in the top 10 across plant-plant interactions in the year 2018 (A) and 2019 (B), and across different fertilizer treatments with time (C)。CK, Inorg, and Org represent the treatment under control, inorganic fertilizer and organic fertilizer, respectively; The capital letter B, S, and P represent plant broadleaf specie *Betula albosinensis*, shrub specie *Salix oritrepha*, and conifer specie *Picea asperata*, respectively; BB, SS, and PP refer to intraspecific plant-plant interactions, while SB, SP, and BP refer to interspecific plant-plant interactions.

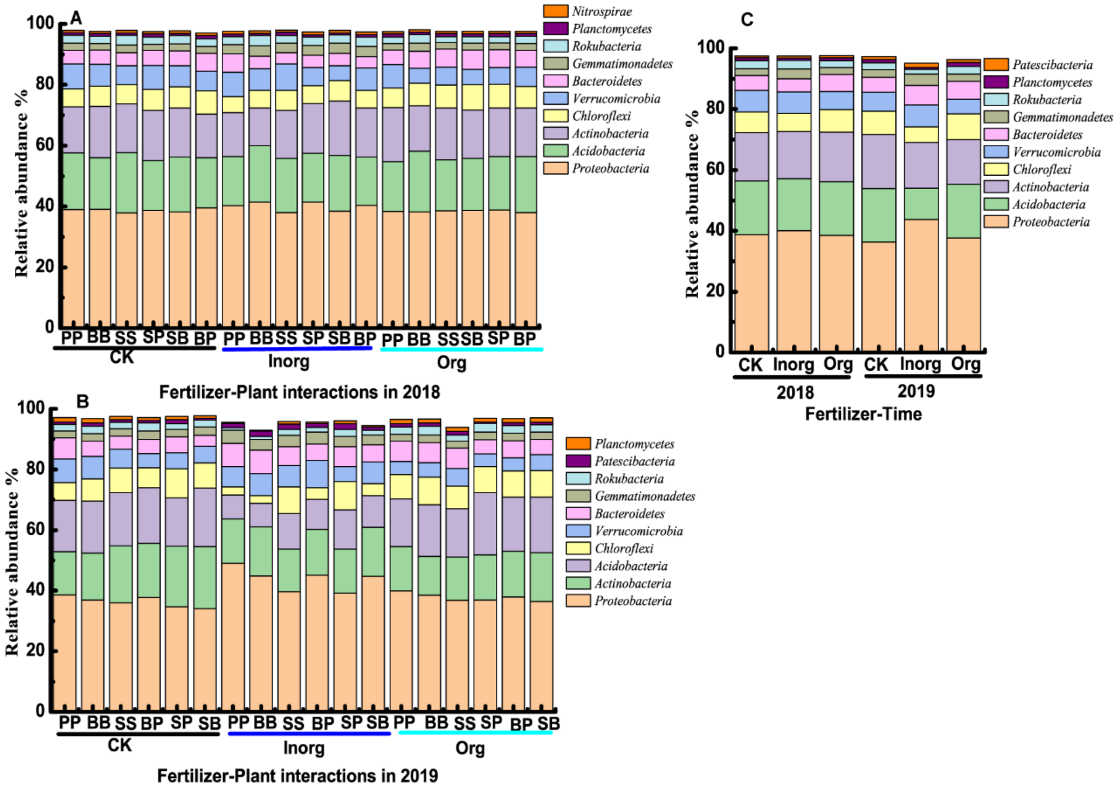

Figure S3. Soil bacterial taxa in 2018yr (A) and 2019yr (B) with different abundance changes under different fertilizers, irrespective of plant-plant interactions (Class: fertilizer treatment; subclass: plant-plant interactions) as detected by LEfSe analysis. The taxa with the absolute LDA scores over 3 and P values less than 0.05 are shown.

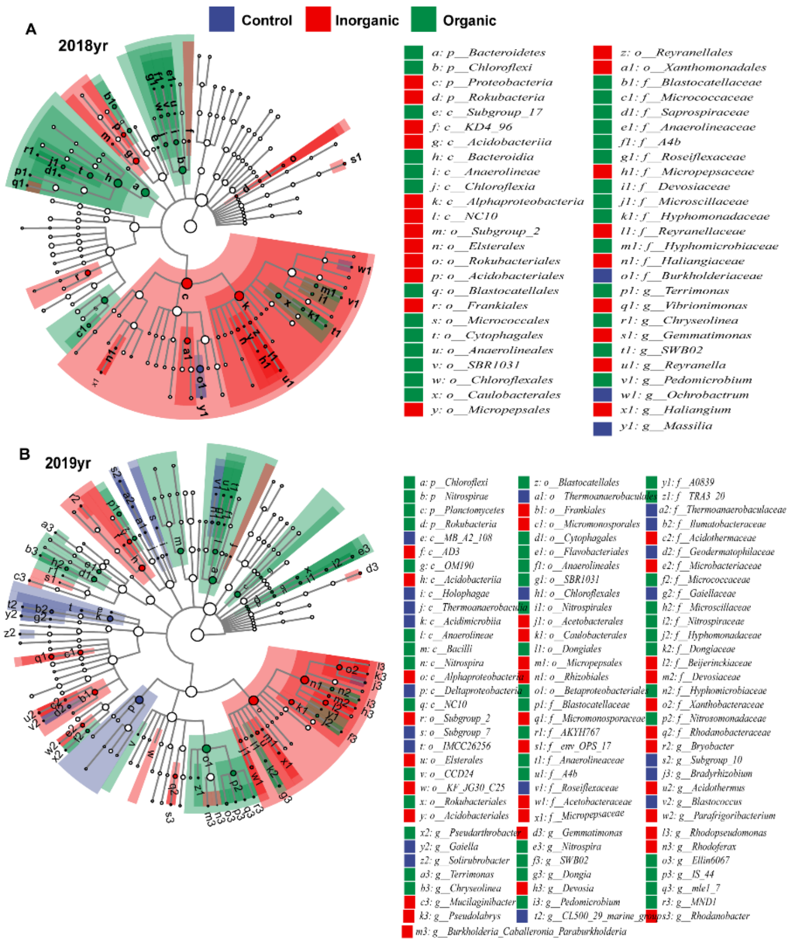

Supplement: Supplementary file 1 [file Data_Sheet_1.pdf]
